# Study on Mechanical Properties and Micro Characterization of Fibre Reinforced Ecological Cementitious Coal Gangue Materials

**DOI:** 10.3390/polym15030700

**Published:** 2023-01-30

**Authors:** Shuai Pang, Xiangdong Zhang, Kaixin Zhu, Jiaze Li, Lijuan Su

**Affiliations:** 1 School of Civil Engineering, Liaoning Technical University, Fuxin 123000, China; 2 Resource Utilization of Coal Gangue and Energy-Saving Building Materials Liaoning Provincial Key Laboratory, Liaoning Technical University, Fuxin 123000, China

**Keywords:** ecological cementitious coal gangue, polypropylene fiber, mechanical properties, numerical simulation, energy consumption characteristics, interface action mechanism

## Abstract

Eco-gelled coal gangue materials (EGCGMs) are usually produced using coal gangue, slag, and fly ash in a highly alkaline environment. Herein, to improve the mechanical properties of such materials, polypropylene fibers were uniformly mixed with them. An unconfined compressive strength test and a three-point bending test of the fiber-reinforced EGCGMs under different conditions were conducted. Based on the performance degradation control technology of the fiber structure, the interface mechanism of the composite materials was analyzed from the micro level using scanning electron microscopy (SEM), energy dispersive spectroscopy (EDS), and X-ray diffraction (XRD). In the mechanical test, the 28 d UCS and flexural properties of the fiber-reinforced EGCGMs were analyzed using the Box–Behnken design response surface design method and orthogonal design method, respectively. The order of significance was as follows: sodium hydroxide, fiber length, and fiber content. Within the scope of the experimental study, when the NaOH content is 3, the fiber content is 5 ‰, and the fiber length is 9 mm, the mechanical properties are the best. Based on the microscopic equipment, it was discovered that the amorphous ecological glue condensation product formed by the reaction of slag and fly ash in the alkaline environment was filled between the coal gangue particles and the fibers, and several polymerization products accumulated to form a honeycomb network topology. The distribution of fibers in the EGCGM matrix could be primarily divided into single embedded and network occurrences. The fiber inhibits the crack initiation and development of the matrix through the crack resistance effect, and improves the brittleness characteristics through the bridging effect during the failure process, which promotes the ductility of the ecological cementitious coal gangue matrix.The results presented herein can provide a theoretical basis for improving the mechanical properties of alkali-activated geopolymers.

## 1. Introduction

Coal is the most abundant and widely distributed fossil fuel on earth. During mining, considerable amounts of coal gangue solid wastes are discharged. Accordingly, China has accumulated more than six billion tons of coal, covering an area of more than 200,000 mu. In addition to its significant land occupancy, long-term coal storage can also cause spontaneous combustion and pollution of the atmosphere and groundwater [1,2]. Blast furnace slag is an industrial waste slag produced by smelting metal iron and perennial open-air storage, which not only occupies a large amount of land, but also causes severe pollution owing to dust and heavy metal ions discharged through rainwater leaching into the atmosphere, soil, and groundwater [3]. Fly ash refers to the fine ash collected from flue gas after coal combustion. A copious amount of fly ash produces dust and pollutes the atmosphere if left untreated. Furthermore, if fly ash is discharged into the water system, it can cause river siltation and pose a threat to the human body and the ecosystem [4,5].

The comprehensive utilization of coal gangue is a long-term technical and economic policy that has received significant attention from researchers. Consequently, several studies focusing on coal gangue have been carried out worldwide, and coal gangue is used as a production building material [6,7,8] in the reclamation of coal gangue and in the backfill of mine goaf [9,10,11,12]. The components of coal gangue are mainly organic carbonaceous and inorganic aluminosilicate minerals, which can be used as siliceous or aluminum raw materials. Recently, certain scholars have used alkali activation technology to activate coal gangue [13,14,15,16]. Coal gangue is used as an aggregate. Solid wastes, such as slag and fly ash, are also depolymerized and repolymerized under alkali activation to prepare high-strength and high-performance geopolymer concrete [17,18,19]. Zhang et al. [20] used red mud and fly ash as silica-alumina raw materials to prepare alkali-activated geopolymer materials under normal temperature curing conditions. They discovered that the 28-d compressive strength reached 21.3 Mpa. Fernández-Jiménez et al. [21] discovered that with the diffusion of an alkali solution from the surface of the fly ash vitreous body to its interior, the generated aluminum silicate gel gradually deposited on the exterior and inside of the vitreous body and encapsulated the unreacted part of the vitreous body to form a dense geopolymer gel; alkali-activated polymers behave similarly to conventional concrete in that they are dense. Although the compressive strength is high, their tensile strength and fracture toughness are poor [22,23,24], resulting in evident brittle characteristics when the polymer is destroyed. These drawbacks seriously hinder the practical engineering applications of alkali-activated polymer materials.

A certain number of fibers, such as carbon fiber, steel fiber, polyvinyl alcohol (PVA) fiber, and sweet sorghum fiber, can be incorporated into geopolymers to not only improve the flexural strength of geopolymers, but also significantly enhance their brittleness and toughness. Farooq Azam Khanzada [25] found that polypropylene fiber can improve the deformation capacity of precast aggregate concrete and prevent the formation and expansion of micro cracks. Oldrich Sucharda [26] proved the positive effect of fiber in concrete by three-point bending test, and the residual tensile strength after peak load was obvious. However, systematic research on the influence of the distribution of polypropylene fibers in a geopolymer matrix on its mechanical properties and microstructure is still lacking. In this study, polypropylene fibers were added to alkali-activated polymer materials based on the design concept of high-ductility, cement-based composite materials. Through the use of macro-indoor unconfined compressive strength tests, three-point bending tests, scanning electron microscopy (SEM), and energy dispersive spectroscopy (EDS) micro-scanning, as well as X-ray diffraction (XRD) techniques, the effects of sodium hydroxide content, fiber length, and fiber content on the mechanical properties of eco-cemented coal gangue materials were revealed at a cross-scale, and an explanation is provided for the interface mechanisms of fiber-reinforced eco-cemented coal gangue materials. The research results can expand the application scope of solid waste, such as fly ash and coal gangue, in the engineering field and provide theoretical and technical support.

## 2. Materials and Methods

### 2.1. Testing Material

**Figure 1:**
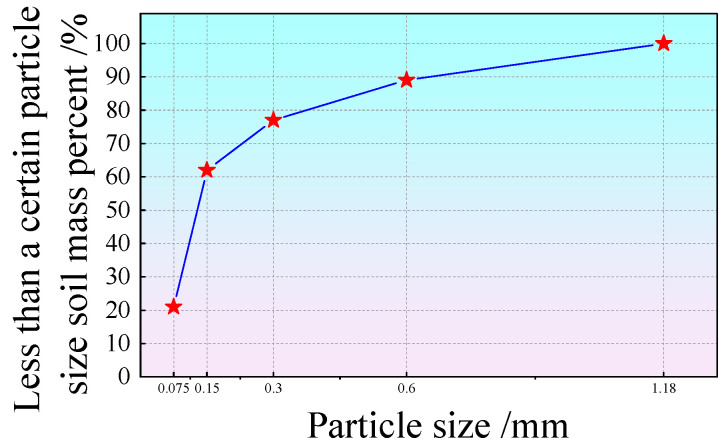
Particle gradation curve of coal gangue.

**Figure 2:**
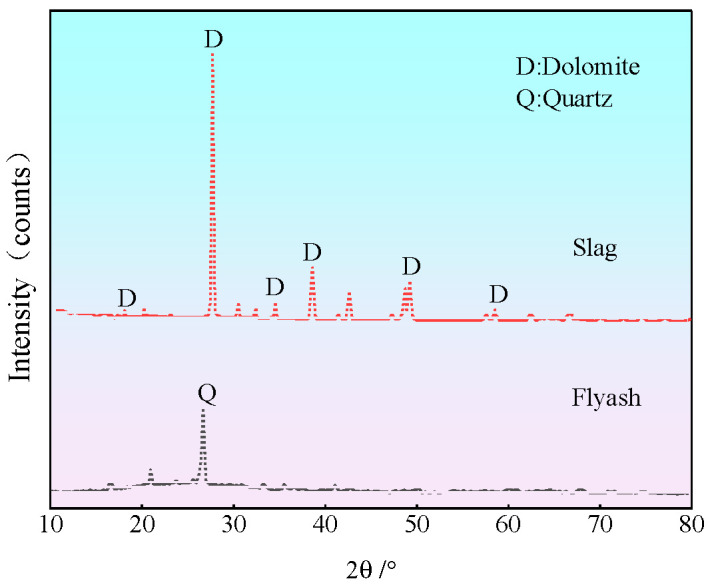
XRD patterns of slag and fly ash.

**Figure 3:**
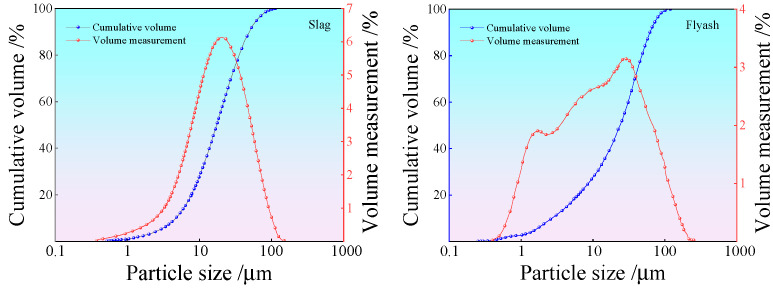
Particle size curve of slag and fly ash.

**Figure 4:**
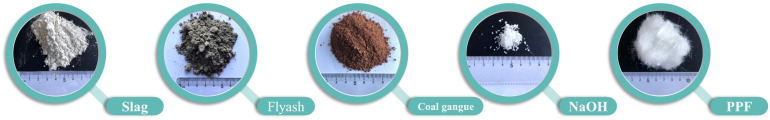
Test raw materials.

The solid raw materials used in the test were solid wastes, including coal gangue, fly ash, and slag. The liquid raw material used was tap water from Fuxin City, and the alkali activators were sodium hydroxide particles. The spontaneous combustion coal gangue was provided by Fuxin Mining Group. The main chemical components and physical indicators are shown in Table 1 and Table 2, respectively. Before the test, the coal gangue was inserted into a mechanical grinder to obtain the fine particle size of solid waste spontaneous combustion coal gangue powder. The particle size was below 1.18 mm, and the particle size distribution curve is shown in Figure 1. Fly ash was procured from the Rizhao Huaneng Power Plant, and blast furnace slag was purchased from Henan Yuanheng Environmental Protection Engineering Co. Ltd. The chemical composition was tested using X-ray fluorescence spectroscopy (XRF). The results are shown in Table 3. The physical and mechanical properties of the slag are summarized in Table 4. The phase structure information of the slag and fly ash was obtained using a Japanese Rigaku bench XRD (MiniFlex 600). The diffraction pattern is shown in Figure 2. The particle sizes of the slag and fly ash were tested using a laser particle size distribution instrument, Bettersize-2000. The particle size curve is shown in Figure 3. Polypropylene fiber was procured from Langfang Tuosheng Insulation Material Co., Ltd. Its physical and mechanical parameters are summarized in Table 5. The raw materials used in the test are shown in Figure 4.

### 2.2. Test Method

In the macroscopic mechanical test, the Box–Behnken design (BBD) response surface optimization was carried out by a design expert, and an orthogonal design assistant was used for the orthogonal experimental design. On the basis of Sun [27] research, this paper sets the slag content as 15%, the coal gangue content as 35%, the fly ash content as 50%, and the water-binder ratio as 0.4. The effects of sodium hydroxide content, fiber content, and fiber length on the compressive and flexural properties of the fiber-reinforced EGCGM were analyzed. The test factor levels of the compressive performance test and flexural performance test are shown in Table 6. In Table 6, A represents the amount of NaOH, %; B represents the amount of fiber, ‰; C represents the length of the fiber, mm. In the flexural performance test, some researchers used the four-point bending test [28,29,30,31,32,33], but the four-point bending test process has a complex clamping structure, while the three-point bending test is simple and practical and can test the region of interest of the sample. Therefore, this test carries out three-point bending test on the sample. In the subsequent exploration process, the author will carry out four-point bending test with more uniform bending moment distribution.

**Figure 5:**
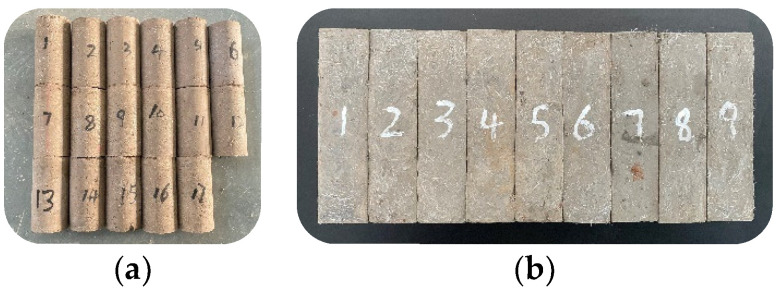
Composite material samples. (**a**) Uniaxial compression test samples (**b**) Three-point bending test sample.

For sample preparation, the raw materials used were weighed and mixed according to the test design quality mix ratio, and a glass rod was used to stir the contents at a constant speed for 3 min; water and sodium hydroxide were mixed and stirred. When the liquid was cooled to room temperature, it was poured into the solid mixture and quickly stirred for 3–5 min. After the slurry had a certain fluidity, the polypropylene fiber was added evenly, and the mixture was stirred for 5–8 min. After stirring, it was poured into a cylindrical mold with a diameter of 50 mm and height of 100 mm and a rectangular mold with a width of 40 mm, height of 40 mm, and height of 160 mm. After vibrating and compacting on a vibration table, the resultant mixture was placed in a standard curing box (the temperature was maintained at 20 °C ± 2 °C and the humidity at approximately 90%), and the mold was demolded after curing for 24 h. After the molding was completed, the curing box was allowed to stand for 28 d. Three parallel samples were prepared for each group; some samples are shown in Figure 5.

**Figure 6:**
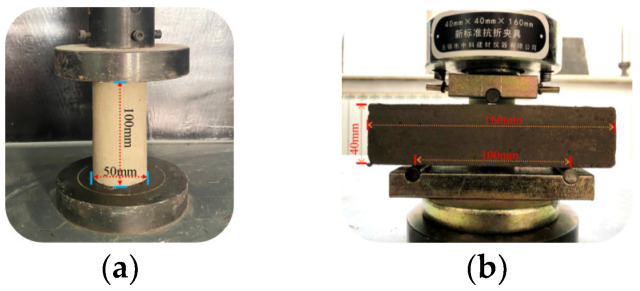
Test loading process. (**a**) Uniaxial compression test process (**b**) Three-point bending test process.

During the tests, according to the “test method for flexural properties of oriented fiber-reinforced polymer matrix composites” (GB/T 3356-2014) [34], the WDW-100 E microcomputer-controlled electronic universal testing machine was used to conduct the unconfined compressive strength and three-point bending tests on samples that had reached the curing age. The loading process is shown in Figure 6. The loading rate was controlled at 0.5 mm/min, and the maximum pressure value at the time of failure of the sample was recorded. When the load decreased by 30% of the maximum load, the test was terminated.

**Figure 7:**
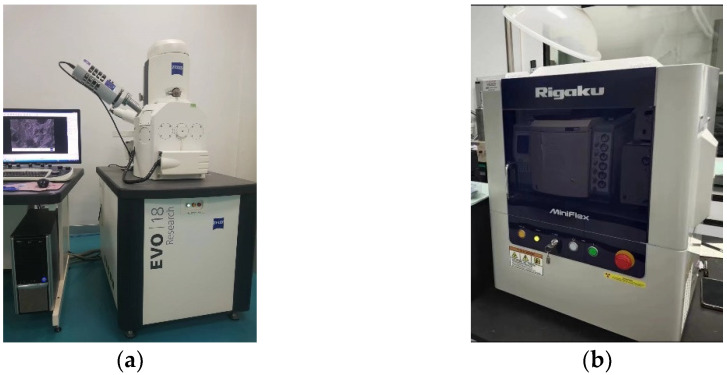
(**a**) Low vacuum scanning electron microscope equipment and (**b**) X-ray diffraction instrument.

After the three-point bending test, the microstructure of some samples was observed using a field emission scanning electron microscope (Zeiss MELIN Compact), as shown in Figure 7a. When a typical area was observed, the equipment was switched from a low to a high magnification mode, the local area was enlarged, and representative images were selected for photographing and preservation. The occurrence state of the fiber in the ecological cementitious coal gangue filling material was expounded, and the degradation control technology of fiber structure performance was applied. The XRD analysis of the fiber-reinforced EGCGM was carried out using a Nippon Rigaku desktop XRD (MiniFlex 600). The diffraction pattern and internal product structure of the EGCGM were analyzed. The equipment is shown in Figure 7b.

## 3. Results and Analysis

### 3.1. Unconfined Compressive Strength Test

#### 3.1.1. Test Results

Based on the BBD response surface method, 17 mix ratio tests were carried out. The test results are shown in Table 7.

#### 3.1.2. Test Result Analysis

A second-order polynomial mathematical model with interaction terms was used to perform multiple regression analysis on the data displayed in Table 7 to obtain a response surface model. Note that the second-order model is often used for response surface function fitting, and its form is shown in Equation (1).

(1) $$Y=\beta_{0}+\sum_{i=1}^{k}\beta_{i}x_{i}+\sum_{i=1}^{k}\beta_{ii}x_{ii}^{2}+\sum_{i=1}^{k-1}\sum_{j=i+1}^{k}\beta_{ij}x_{i}x_{j}+\varepsilon$$

where Y is the response value, x is the influence factor, β is the coefficient, ε is the error value, and k is the level number of variables.

The response surface function of the fiber-reinforced EGCGM 28d UCS was obtained as follows:

(2) $$\begin{array}{l} Y=-4.96475+1.999\cdot x+3.46\cdot y+0.077167\times 6+0.0025\cdot x\cdot y\\ +0.010833\cdot x\cdot 6-0.19167\cdot y\cdot 6-0.10225\cdot x^{2}-0.34225\cdot y^{2}+0.003083\times 36 \end{array}$$

**Figure 8:**
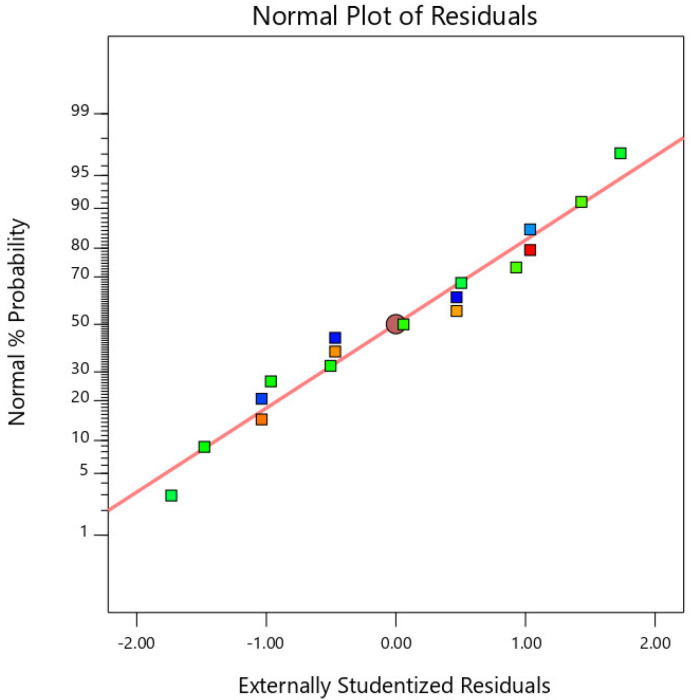
Normality test diagram.

The $R^{2}$ value for the fiber-reinforced EGCGM 28d UCS model was 95.82%. Notably, the closer the model $R^{2}$ is to one, the higher the fitting degree of the model. Therefore, 95.82% of the test results in this experiment can be explained through multiple nonlinear regression analysis, and the fitting degree of the model is higher. Here, C.V. represents the degree of dispersion of the model data, which is not greater than 0.15. Here, the C.V. value is 0.0192, the test data credibility is high, and the test results conform to the normal distribution, as shown in Figure 8.

The variance analysis results of the fiber-reinforced EGCGM 28d UCS response surface model are presented in Table 7. The value of the response surface regression model is 0.05, indicating that the regression model fitting analysis results have high reliability. Table 8 shows the significance of every single factor on the fiber-reinforced EGCGM 28d UCS model, ranked from large to small: sodium hydroxide content > fiber length > fiber content.

**Figure 9:**
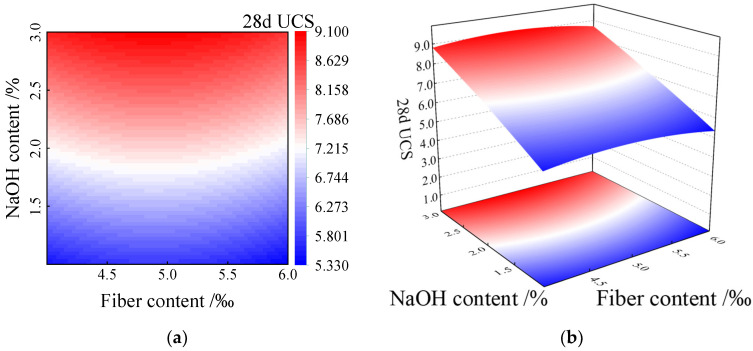
Interaction diagram of factors A and B on 28 d UCS. (**a**) Contour map of 28 d UCS of factors A and B. (**b**) Response surface diagram of factors A and B to 28 d UCS.

The response surface regression model of 28 d UCS was used to draw the contour map and response surface map of factors A and B, respectively, as shown in Figure 9.

As can be observed from Figure 9, in the experimental study range, the 28 d UCS progressively increased with an increase in the sodium hydroxide content (factor A), while the fiber content (factor B) also increased in the process. The 28 d UCS change trend demonstrated an increase, followed by a decrease. Simultaneously, the influence of the two factors on the 28 d UCS had an interaction, and the response surface graph in Figure 9b appeared owing to the bending phenomenon.

**Figure 10:**
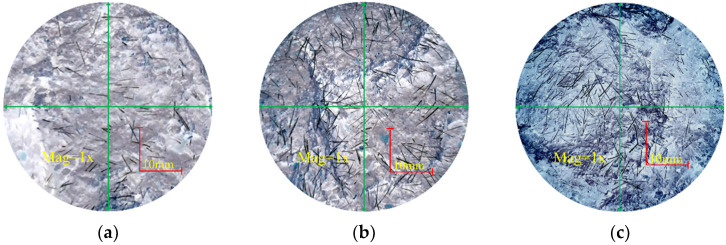
The failure cross section of samples with different fiber content. (**a**) Sample 5 (4%). (**b**) Sample 10 (5%). (**c**) Sample 13 (6%).

Figure 10 shows the cross-section morphology of the specimens with different fiber contents. The processed images shown in Figure 10a–c were obtained using image-processing methods, such as noise reduction, enhancement, segmentation, and recognition and measurement using the image-processing pipeline digital image-processing technology. The characteristic data of the cross-section fiber distribution after processing can be clearly observed.

When the fiber content is 4%, as shown in Figure 10a, the fiber is randomly distributed in the ecological cementitious coal gangue matrix in the form of an inlay and in other forms, and the degree of dispersion is high. In the alkaline environment, the fiber is gradually wrapped by the solid waste polymerization product during the polymerization reaction process. Then, the gripping effect occurs. The gripping area increases with increasing fiber embedding length. The fiber is in a tensile state when it deforms or is destroyed under the external load. The fiber and coal gangue particles are bound by friction and bonding forces, which play a role in limiting relative sliding, connecting, and reducing the relative displacement. When the fiber content is 5%, the degree of overlap between the fibers increases, and a three-dimensional network structure with random distribution is formed inside the mixed system. As shown in Figure 10b, the fiber is bonded with the ecological cementitious coal gangue matrix. A sample subjected to an axial load can not only share the stress effect with the ecological cementitious coal gangue matrix, but can also effectively limit coal gangue particle dislocation, reduce the overall deformation of the blending material, and reduce the strain value of the sample to reach failure strength. When the fiber content is increased to 6%, the fiber is unevenly distributed in the eco-cemented coal gangue matrix. Given the large degree of fiber overlap, superposition, agglomeration, and winding occur. Finally, a defected heterogeneous body with weak cross-section is formed, which destroys the integrity of the eco-cemented coal gangue aggregate. Therefore, the fiber cannot effectively exert a high tensile force, thereby degrading the compressive performance of the eco-cemented coal gangue matrix.

### 3.2. Three-Point Bending Tests

#### 3.2.1. Test Result

By considering the influence of sodium hydroxide, the fiber content, and the fiber length on the flexural performance of the ecological cementitious coal gangue, based on the principle of mathematical statistics and orthogonality, an orthogonal test was carried out according to the principle of “balanced dispersion” and “neat and comparable,” and the range and variance were used in conjunction with each other to conduct a scientific and effective analysis of the test factors.

The load corresponding to the peak point on the loading curve in the three-point bending test was recorded as the maximum load that the sample could bear. According to the basic theory of elastic mechanics, the formula for calculating the flexural strength of composite materials is as follows:

(3) $$f_{f}=\frac{3FL}{2bh^{2}}$$

where $f_{f}$ is the flexural strength of the sample, MPa; F is the failure load of the specimen, N; L is the span between supports, mm; b is the width of the sample section, mm; and h is the height of the sample section, mm.

The orthogonal test results are shown in Table 9, wherein the blank column shows the error analysis values.

#### 3.2.2. Test Result Analysis

**Figure 11:**
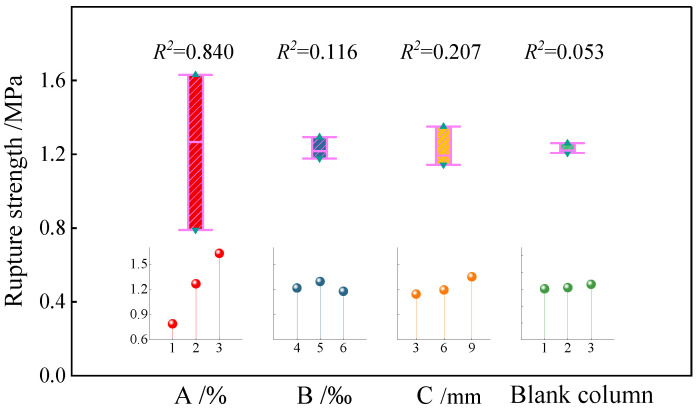
Influence of various influencing factors on the average flexural strength.

The range of the mean value of the flexural strength of the sample at each factor level was calculated through the test results. To better intuitively reflect the influence degree of each factor, the level of each factor was used as the abscissa, and the mean value of the flexural strength of the sample was used as the ordinate to construct the mean effect diagram of the flexural strength, as shown in Figure 11 (the curve shown in Figure 11 is depicted by nine sets of test results, which only represents the trend between the two factors). The calculation formula of the range R is as follows:

(4) $$R=\max\left({\bar{R}}_{c}\right)-\min\left({\bar{R}}_{c}\right)$$

where R is the range, and ${\bar{R}}_{c}$ is the mean value of the failure strength at each factor level.

According to the value of *R* in Figure 11, the order of influence of various factors on the flexural strength of the fiber-reinforced EGCGM is as follows: sodium hydroxide content > fiber length > fiber content. The flexural strength of the sample increases with the increase in sodium hydroxide content and reaches the local maximum when the content is 3%. The flexural strength of the sample first increases and then decreases with the increase in fiber content. When the content is 5%, the flexural strength of the sample reaches the local maximum. The flexural strength of the sample increases with the increase in fiber length and reaches the local maximum when the length is 9 mm. Therefore, in the range analysis, the local optimal level combination is A3C3B2, and the optimal local combination of the test is as follows: sodium hydroxide content = 3%, fiber length = 9 mm, and fiber content = 5%.

A significance test of the orthogonal test results was carried out via variance analysis. Table 10 shows the variance analysis results of different factors on the flexural strength of the samples. According to the significance test results summarized in the table, the order of influence is as follows: sodium hydroxide content > fiber length > fiber content, which is consistent with the results of the range analysis. Furthermore, according to the relationship between the *F*-ratio and *F*-critical value, the effect of the sodium hydroxide content on the flexural strength of the sample is highly significant.

### 3.3. Analysis of Sample Energy Consumption Characteristics

**Figure 12:**
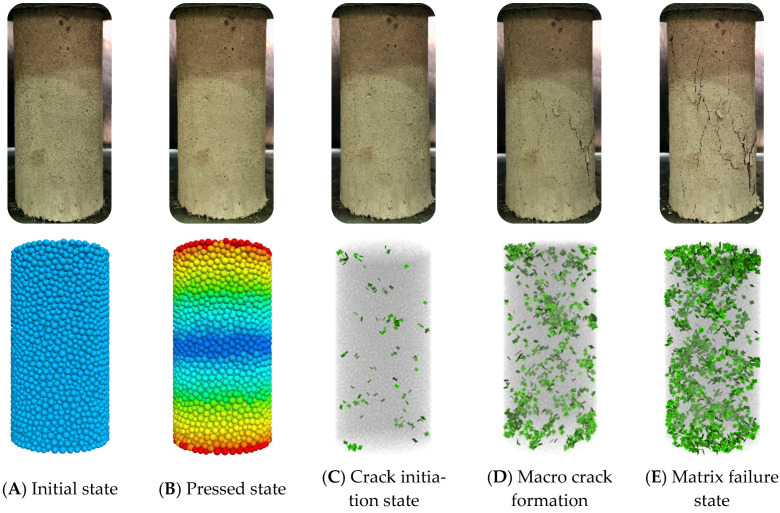
Failure evolution process of the specimen.

Typically, fiber-reinforced EGCGM yield failures and damage are essentially caused by energy dissipation and release. This energy evolution occurs throughout the process of specimen deformation and failure. The three-dimensional particle flow numerical simulation software (PFC^3D^) was used to establish a uniaxial compression standard sample with a diameter of 50 mm and a height of 100 mm, as shown in Figure 12. During the loading process, the control conditions of the PFC numerical test were consistent with the loading conditions of the indoor triaxial compression test. Based on the indoor unconfined compressive strength test results, the approximation method was used to calibrate the mesoscopic parameters required in the discrete element numerical simulation process. The main parameters involved in the PFC3D numerical model are shown in Table 11.

The numerical simulation of the failure mode evolution process of the fiber-reinforced EGCGM specimen is shown in Figure 12.

With the increase of axial load, the failure process of the specimen is divided into four stages, as shown in Figure 12. The first stage: the elastic deformation of the sample matrix occurs, and the overall failure behavior does not occur, which is defined as the sample compaction stage; the second stage: defined as the micro-crack initiation stage of the sample; the third stage: the development and expansion of microcracks, which indirectly leads to the deterioration of macroscopic mechanical properties, is defined as the macroscopic crack inoculation stage of the sample. The fourth stage: the fiber is strengthened in the aggregate, and the slip or fracture phenomenon is gradually produced. The failure behavior of the sample matrix is defined as the overall failure stage of the sample.

For uniaxial compression behavior, the energy dissipation of the sample can be mainly divided into the energy consumed by the damage and plastic deformation of the mixed material and the released strain energy stored in the matrix; that is, the sample absorbs the energy input from the outside to deform the skeleton of the coal gangue particles and hinder the development of micro-defects and crack propagation and slip.

From the energy perspective, when considering the deformation of a unit volume of the sample element under the action of an external force, the sample element is assumed to be a closed system, and no loss of heat exchange energy occurs with the outside world during deformation and failure. The total energy U input by the external load to the rock mass is entirely converted into the elastic strain energy $U^{e}$ and unit dissipation energy $U^{d}$ that the unit can release. The first law of thermodynamics indicates the following:

(5) $$U=U^{e}+U^{d}$$

Under uniaxial compression, the energy calculation formula of the sample is as follows.

(6) $$U=\int_{0}^{\varepsilon }\sigma d\varepsilon$$

(7) $$U^{e}=\frac{1}{2E_{u}}\sigma^{2}$$

(8) $$U^{d}=\int_{0}^{\varepsilon }\sigma d\varepsilon -\frac{1}{2E_{u}}\sigma^{2}$$

The energy loss ϖ of the specimen element during compression is defined as follows:

(9) $$\varpi =\frac{U^{d}}{U^{c}}$$

where $U^{c}$ is the critical energy dissipation value when the element strength is lost, which is a material constant and independent of the stress state. When $U^{c}$ is equal to $U^{d}$, ϖ is equal to one, that is, the material strength is lost.

Combined with Equations (5)–(9), the Einstein summation convention is used and simplified to obtain the following:

(10) $$U^{c}=\int_{0}^{\varepsilon }\sigma d\varepsilon -\frac{1}{2}\sigma \varepsilon^{e}$$

Equation (10) is the fiber-reinforced EGCGM unit strength loss criterion based on energy dissipation.

The unit dissipation energy $U^{d}$ is used to form the internal damage and plastic deformation of the unit, and its change satisfies the second law of thermodynamics, that is, the change in internal state conforms to the trend of entropy increase.

According to the test results of the unconfined compressive strength test, the energy parameters of the sample under peak stress are calculated, as shown in Table 12.

**Figure 13:**
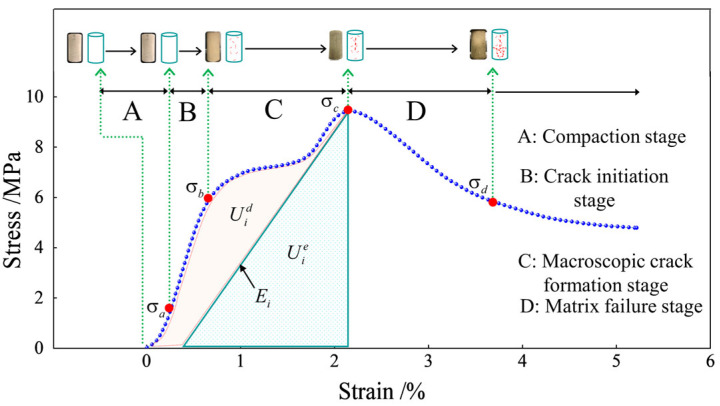
Evolution process of specimen failure energy.

Figure 13 shows the stress–strain curve of the fiber-reinforced EGCGM specimen element. The area $U_{i}^{d}$ represents the energy consumed by the damage and plastic deformation of the element, and the shaded area $U_{i}^{e}$ represents the releasable strain energy stored in the element.

Notably, energy conversion is the essential feature of material physical processes, and material damage is a state instability phenomenon driven by energy. In the initial stage of energy input, when the stress increases from zero to $\sigma_{a}$, most of the energy is absorbed by the sample. Concurrently, the energy is mainly used for the compaction of the pores and holes between the medium particles in the sample matrix and the elastic deformation of the coal gangue particle skeleton. This process is the compaction stage of the fiber-reinforced EGCGM sample under the initial load. With further input of external load energy, the stress increases from $\sigma_{a}$ to $\sigma_{b}$. When the input energy is greater than the limit that the elastic deformation of the sample matrix can withstand, the excess energy is mainly used for the development, evolution, slip, and strength degradation of micro-cracks inside the sample, which directly leads to the increase in the strength of the sample matrix. Slowing down, when the stress increases to $\sigma_{c}$, macroscopic crack formation occurs in the sample; with the continuous input of energy, the damage to the sample matrix continues to aggravate. At this stage, the overall bearing capacity begins to decrease, and the stress decreases from $\sigma_{c}$ to $\sigma_{d}$. However, the fibers undergo a jumping process of adhesion and sliding in the aggregate and gradually slip and fracture. When the total input energy exceeds the dissipated energy required for the failure of the fiber-reinforced EGCGM specimen matrix, internal cracks develop, penetrate each other, and form macroscopic cracks. At this point, energy release occurs, which directly leads to specimen failure. This is consistent with the conclusion of numerical simulation.

## 4. Microscopic Characterization of the Composite Materials

### 4.1. Overall Morphology Analysis

The microstructures of different samples after the three-point bending test were observed via SEM. When the typical area was observed, the low-power mode was switched to the high-power mode, and the local area was enlarged. The representative images were captured and saved. The magnification range of the SEM images obtained in this experiment was 50× to 30,000×.

**Figure 14:**
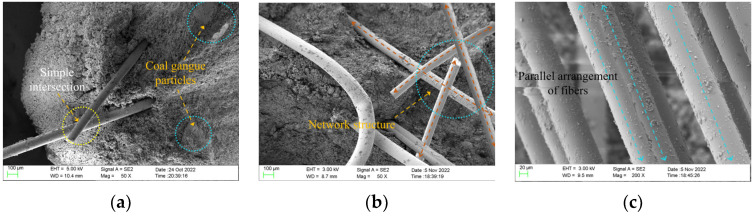
The micro-morphology of samples with different fiber content. (**a**) Sample 5 (4%). (**b**) Sample 10 (5%). (**c**) Sample 13 (6%).

Through the unconfined compressive strength test and the three-point bending test, we discover that the incorporation of polypropylene fiber into the ecological cementitious coal gangue matrix can effectively improve the mechanical properties of the ecological cementitious coal gangue filling material, and the incorporated fiber has an optimal reinforcement ratio of 5%. When the fiber content is less than the optimal reinforcement ratio, as shown in Figure 14a, the lap density between the fibers and the fibers in the matrix is small, and the fibers are freely distributed in the skeleton voids formed by the coal gangue particles. With the increase in curing age, the polymerization behavior product adheres to the fiber surface and produces a more evident gripping effect when subjected to a load. With the increase in fiber content, as shown in Figure 14b, the fibers are randomly distributed inside the matrix, forming a relatively stable three-dimensional spatial structure system. When the coal gangue particles are staggered and rearranged under the action of an external load, the more stable fiber network structure system constrains their displacement and deformation, thus improving the overall stability of the sample. This allows the external load to be dispersed more evenly throughout the sample, reduces stress concentrations, and distributes the stress.

When the fiber content exceeds 5%, as shown in Figure 14c, the fiber has a low degree of dispersion in the eco-cemented coal gangue matrix and cannot be uniformly dispersed. Consequently, the polymer product cannot entirely contact with the fiber, resulting in a decrease in the fiber holding area. No effective cementation occurs between the entangled fibers in the sample, which makes the matrix loose and reduces the compactness of the entire material. When the specimen is subjected to an external load, the fibers superimposed on the weak layer quickly slip and crack, and the fibers cannot play a reinforcing role. Thus, the overall structure of the specimen is destroyed rapidly.

### 4.2. Ductility-Strengthening Mechanism Analysis

For the fiber-reinforced ecological cementitious coal gangue, the complex composition of the fiber, coal gangue, slag, and fly ash determines the heterogeneity and variability of the physical and mechanical properties of the material. Geopolymer materials have high compressive strength but poor tensile strength and fracture toughness, resulting in evident brittle characteristics upon destruction. The addition of an appropriate amount of polypropylene fiber can further optimize the fracture properties of the EGCGM so that the ecological cementitious coal gangue matrix has a better blocking effect on the later-developed cracks during the fracture process, and the brittle failure is transformed into ductile failure. In this study, the ductility-strengthening mechanism of ecological cementitious coal gangue was revealed via SEM, EDS, and XRD.

In the alkaline excitation solution, covalent bonds, such as the high-energy aluminum–oxygen bond (–Al–O–) and the silicon–oxygen bond (–Si–O–) in the aluminosilicate material, are broken by ${\mathrm{OH}}^{-}$ ions, and the freely moving Si monomer $\left({\mathrm{Si}(\mathrm{OH})}_{4}\right)$ and Al monomer $\left({\mathrm{Al}(\mathrm{OH})}_{4}^{-}\right)$ are released from the surface of the raw material phase. The dissolved Si and Al monomers are reconstructed by dehydroxylation and reacted with alkali metal ions to form initial polymers such as silicate and aluminosilicate. As the reaction continues, the initial polymer is further condensed to form a three-dimensional network structure composed of $\left[{\mathrm{SiO}}_{4}\right]$ and $\left[{\mathrm{AlO}}_{4}\right]$ tetrahedrons. The polyciliate gel material finally condenses and hardens to form a polymer bulk material.

**Figure 15:**
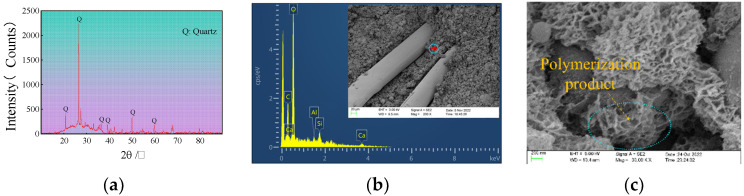
The X-ray spectrum, EDS energy spectrum and microstructure of the reaction products were analyzed. (**a**) X-ray spectrum analysis. (**b**) EDS energy spectrum. (**c**) Microstructure.

The EGCGM can be regarded as a continuous graded particle accumulation system, and the voids between coal gangue particles can be filled by amorphous gel substances formed by slag and fly ash in an alkaline environment. According to the Aim and Goff model, the system exhibits the closest packing when the alkali-activated composite material mixed with an ultrafine admixture is regarded as a multivariate system. The slag in the EGCGM is finer than fly ash. After replacing a portion of the fly ash with slag, the amorphous slag particles filled in the pores between the spherical fly ash particles, resulting in a denser filling structure and a meso-level self-compact packing system. As shown in Figure 15, when the EGCGM is cured for 28 d, numerous polymerization products accumulate to form a honeycomb mesh topology. The honeycomb topology is a continuously changing structure between the solid and hollow structures. When resisting external forces, the structure can disperse and bear external forces from all directions, and it has good structural stability and uniform buffer strength.

**Figure 16:**
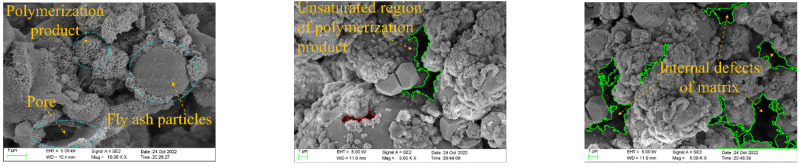
Pore defects between hydration products inside the material.

As a deformation aggregate containing continuously distributed defects, in the early stage of damage, defects, such as micro-cracks and micro-voids inside the eco-gelled coal gangue matrix, are random. Owing to the evaporation of pore water from the sample during setting and hardening, pores are left vacant, resulting in an unsaturated zone of the polymerization product (Figure 16). When the randomly distributed nano-pores are connected, they form micro-cracks within the system, and the original micro-cracks are responsible for the failure of the ecological gelled coal gangue matrix. Conversely, micro-cracks contribute to the initiation of macro-cracks; they exert a dual effect of shielding and deterioration of the main cracks. The fiber can prevent the induction of these micro-cracks, thereby reducing the number of crack sources and making the crack scale smaller. Therefore, during the stressing process of the sample, the fiber inhibits the germination and expansion of the cracks and produces a crack resistance effect in the ecological cementitious coal gangue matrix.

**Figure 17:**
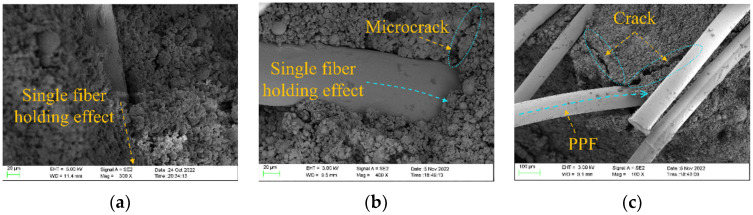
Mechanism diagram of fiber action inside the sample. (**a**) Gripping effect. (**b**) Gripping effect. (**c**) Inhibition of fission development.

As shown in Figure 17a, owing to the binding effect between the fibers and the products of the geopolymerization reaction, the formed crack is immediately blocked when it is adjacent to the fibers, thereby preventing the crack from continuing to expand during specimen failure, as shown in Figure 17b. As the fibers form a uniform random support system inside the specimen, an effective secondary strengthening effect is produced, as shown in Figure 17c. Simultaneously, when the random arrangement of fibers in the sample system constitutes a three-dimensional spatial network structure, the polymerization products generated by coal gangue particles, slag, and fly ash in an alkaline environment are woven or interlocked into a unified coherence matrix. Consequently, when the fiber is subjected to an initial load, the fiber inhibits the initiation of cracks in the sample matrix and the development of primary cracks to sub-micro-cracks, improving the mechanical properties of the ecological cementitious coal gangue matrix. With the increase in load, the micro-cracks in the ecological coal gangue matrix gradually form sub-microcracks and enter the stable expansion stage. The polypropylene fibers distributed randomly at the crack tip begin to play an anchoring role. When the crack tip develops infinitely close to the fiber, the high-stress concentration around the crack tip causes the fiber around the crack tip to separate from the interface of the ecological cementitious coal gangue matrix. As the load is increased, cracks pass through the fiber, causing macroscopic cracks to expand. When the cracks pass through the fiber, the sample enters a state of instability and failure, and the number of crack expansions increases sharply. The shear and tensile cracks together form macroscopic cracks in the sample, and the matrix is disconnected, thus producing penetrating cracks. The interface separation between the fiber and the cementitious coal gangue matrix results in a strain mutation characterized by an interface slip phenomenon or pull-off. In this instance, the polypropylene fiber microscopically creates a bridging effect. Macroscopically, the ductility of the ecological cementitious coal gangue matrix is enhanced.

## 5. Conclusions

The mechanical properties and interface mechanisms of fiber-reinforced EGCGMs were studied via a macroscopic unconfined compressive strength test, a three-point bending test, and SEM. The following conclusions were derived by analyzing the test results:

(1) Based the BBD response surface design principle and orthogonal test design, the effects of various factors on the 28 d UCS and flexural strength of the fiber-reinforced EGCGM were analyzed, respectively. The order of significance from largest to smallest was as follows: sodium hydroxide content > fiber length > fiber content. In the unconfined compressive strength test: when the NaOH content is 3%, the fiber length is 9 mm, and the fiber content is 5‰, the 28d unconfined compressive strength of the sample reaches the maximum (9.34 MPa). In the three-point bending test, the local optimal combination of the test: the sodium hydroxide content is 3%, the fiber length is 9 mm, and the fiber content is 5‰.
(2) With the help of microscopic scanning technology, it was found that the amorphous eco-gel coagulation products formed by the reaction of slag and fly ash in an alkaline environment were filled between the coal gangue particles and fibers, and several polymerization products accumulated to form a honeycomb network topology. When resisting external forces, the structure could be dispersed to bear external forces from all directions, with good structural stability and uniform buffer strength.
(3) The distribution pattern of fibers in the ecological cementitious matrix could be divided into single embedded and reticular occurrences. When crack initiation and development connection occurred in the matrix, the fiber produced a crack resistance effect by inhibiting the matrix, and by bridging during the failure process, the brittleness of the ecological cementitious coal gangue matrix was enhanced, which promoted its ductility.

In this study, polypropylene fiber was uniformly mixed into the ecological cementitious coal gangue material, and the influence of different factors on the unconfined compressive strength and flexural strength of the sample was explored. Based on SEM, EDS, and XRD, the interface mechanism of composite materials was analyzed from the micro level.

## Figures and Tables

**Table 1:** Main chemical components of spontaneous combustion coal gangue obtained from a mine in Fuxin.

Component	SiO_2_	Al_2_O_3_	Fe_2_O_3_	CaO	MgO	SO_3_	Ignition Loss
Content/%	48.78	21.86	5.38	3.87	0.82	0.16	0.79

**Table 2:** Main physical indexes of spontaneous combustion coal gangue in Fuxin.

Serial Number	Item	Parameter
1	Density grade (kg/m^3^)	1100
2	Compressive strength of concrete cylinder (MPa)	6.0
3	Softening index	0.94
4	Loss of boiling quality (%)	2.3
5	Soil content (%)	0.64
6	Organic content	Up to standard
7	Needle-like content (%)	7.85
8	Tamping density (kg/m^3^)	1550
9	Tap density (kg/m^3^)	1620
10	Crushing value (%)	16.50
11	Water absorption (%)	8.5
12	Apparent density (kg/m^3^)	2077
13	Percentage of void (%)	22

**Table 3:** Chemical constituents wt/%.

Chemical Composition	CaO	Fe_2_O_3_	SiO_2_	Al_2_O_3_	MgO	Na_2_O	K_2_O	SO_3_	FeO	S
Flyash	4.13	3.77	58.79	25.12	0.45	0.98	1.15	0.39	-	-
Slag	44.36	0.38	33.25	13.27	6.31	0.33	0.28	-	0.89	1.1

**Table 4:** Physical property index of blast furnace slag.

Density/g·cm^−3^	Specific Surface Area/m^2^·kg^−1^	Burning Vector/%	Chlorion/%	Mobility Ratio/%	Water Content/%
2.9	428	0.21	0.024	109	0.32

**Table 5:** Physical properties of polypropylene fibers.

Type	Density/g·cm^−3^	Tensile Strength/MPa	Elastic Modulus/GPa	Melting Point/°C	Ignition Point/°C	Fracture Elongation/%	Dispersibility
Bundle monofilament	0.91	≥300	≥3.5	165	590	≥15	Fabulous

**Table 6:** Test factor level.

Level	Factor		
	A	B	C
1	1	4	3
2	2	5	6
3	3	6	9

**Table 7:** Test results.

Sample	NaOH Content/%	Fibber Content/‰	Fibber Length/mm	28 d UCS/MPa
S1	2	6	3	7.11
S2	1	5	9	5.94
S3	1	6	6	5.37
S4	2	4	3	7.21
S5	1	4	6	5.45
S6	1	5	3	5.62
S7	3	4	6	8.78
S8	2	6	9	7.12
S9	2	5	6	7.35
S10	2	5	6	7.4
S11	2	5	6	7.53
S12	2	5	6	7.69
S13	3	6	6	8.71
S14	3	5	3	8.89
S15	3	5	9	9.34
S16	2	4	9	7.42
S17	2	5	6	7.64

**Table 8:** Analysis of variance.

Source	Sum of Squares	df	Mean Square	F-Value	*p*-Value	
Model	22.97	9	2.55	128.98	<0.0001	significant
A-A	22.24	1	22.24	1124.02	<0.0001	
B-B	0.0420	1	0.0420	2.12	0.1883	
C-C	0.1152	1	0.1152	5.82	0.0466	
AB	0.0000	1	0.0000	0.0013	0.9726	
AC	0.0042	1	0.0042	0.2135	0.6581	
BC	0.0132	1	0.0132	0.6683	0.4406	
A^2^	0.0440	1	0.0440	2.22	0.1795	
B^2^	0.4932	1	0.4932	24.92	0.0016	
C^2^	0.0032	1	0.0032	0.1638	0.6977	
Residual	0.1385	7	0.0198			
Lack of Fit	0.0519	3	0.0173	0.7976	0.5561	not significant
Pure Error	0.0867	4	0.0717			
Cor Total	23.11	16				

**Table 9:** Orthogonal test results.

Sample Number	A/%	B/‰	C/mm	Blank Column	Rupture Strength/MPa
F1	1	4	3	1	0.67
F2	1	5	6	2	0.81
F3	1	6	9	3	0.89
F4	2	4	6	3	1.25
F5	2	5	9	1	1.43
F6	2	6	3	2	1.12
F7	3	4	9	2	1.73
F8	3	5	3	3	1.64
F9	3	6	6	1	1.52

**Table 10:** Analysis of variance.

Factor	Square of Deviance	Freedom	*F*-Ratio	*F*-Critical Value	Significance
A	1.065	2	3.669	3.110	*
B	0.021	2	0.072	3.110	
C	0.070	2	0.241	3.110	
Blank column	0.005	2	0.017	3.110	
Error	1.16	8			

**Table 11:** Main meso-parameters involved in PFC^3D^ numerical model.

E/GPa	K_n_/K_s_	μ	n	ρ/kg·m^−3^	ν
23.085	1.0	0.5	0.3	2450	0.2

**Table 12:** Energy parameters of samples under different conditions.

Sample	NaOH Content/%	Fibber Content/‰	Fibber Length/mm	$U/kJ\cdot m^{-3}$	$U^{e}/kJ\cdot m^{-3}$	$U^{d}/kJ\cdot m^{-3}$
S1	2	6	3	8377.59	5290.11	3087.48
S2	1	5	9	7453.62	4931.35	2522.27
S3	1	6	6	6992.14	4212.26	2779.88
S4	2	4	3	8759.03	5672.04	3086.99
S5	1	4	6	7189.29	4398.15	2791.14
S6	1	5	3	7301.58	4479.29	2822.29
S7	3	4	6	11,218.66	8189.14	3029.52
S8	2	6	9	8476.34	6257.31	2219.03
S9	2	5	6	9421.59	6405.29	3016.3
S10	2	5	6	9537.68	6472.17	3065.51
S11	2	5	6	9508.41	6453.27	3055.14
S12	2	5	6	9471.68	6440.19	3031.49
S13	3	6	6	10,957.22	7450.94	3506.28
S14	3	5	3	11,492.09	8274.33	3217.76
S15	3	5	9	12,870.78	10,156.53	2714.25
S16	2	4	9	9153.85	6199.84	2954.01
S17	2	5	6	9379.61	6398.52	2981.09

## Data Availability

Due to data privacy, authors have no right to share data.
